# Recipient-driven and donor-driven determinants of delayed graft function in paired kidney transplantation: evidence from a Chinese cohort

**DOI:** 10.1080/0886022X.2026.2717468

**Published:** 2026-08-28

**Authors:** Xuyuan Zhu, Yu Zhang, Yuxiang Chen, Shanda Li, Kun Wang, Tao Li, Xiaojie Ma, Hongtao Jiang

**Affiliations:** a Department of Kidney Transplantation, the Second Affiliated Hospital of Hainan Medical University, Haikou, China; b Department of Rehabilitation, the Second Affiliated Hospital of Hainan Medical University, Haikou, China

**Keywords:** Delayed graft function, kidney transplantation, risk factors, Donor-Recipient mismatch, paired kidney analysis

## Abstract

**Background:**

Delayed graft function (DGF) is a frequent complication of kidney transplantations. When both kidneys from a single deceased donor are transplanted into two recipients, DGF outcomes may be discordant, affecting only one recipient, or concordant, affecting both recipients. The factors underlying these within-donor differences, particularly in the Chinese population, remain poorly understood.

**Methods:**

We retrospectively analyzed 356 deceased-donor kidney transplants performed in our hospital between 2018 and 2022. We first examined recipient-specific factors associated with discordant DGF among paired recipients from the same donor. Second, we assessed donor factors associated with concordant DGF in both recipients.

**Results:**

In discordant pairs, multivariate regression identified recipient body mass index (BMI; odds ratio [OR] = 1.22, per unit changes; 95% CI 1.03–1.44; *p* = 0.019), dialysis duration (OR = 1.31, per years; 95% CI 1.01–1.69; *p* = 0.041), and number of HLA mismatches (OR = 1.47, 95% CI 1.06–2.02; *p* = 0.019) as independent predictors of DGF. Concordant analyses comparing donor pairs with DGF in both recipient and donor terminal creatinine (OR = 1.03, per mg/dL; 95% CI 1.01–1.05; *p* = 0.001), duration of donor hypertension (OR = 2.40, per years; 95% CI 1.52–3.81; *p* < 0.001), and donor warm ischemia time (OR = 1.67, per hours; 95% CI 1.15–2.44; *p* = 0.007) were independently associated with DGF in both recipients.

**Conclusions:**

Among kidney recipients from the same donor, higher BMI, longer dialysis duration, and greater HLA mismatch independently predicted DGF when outcomes were discordant. Conversely, donor-related factors, higher terminal creatinine, longer hypertension duration, and prolonged warm ischemia drove DGF in both recipients.

## 1. Introduction

Chronic kidney disease (CKD) has escalated into a defining global health crisis, currently affecting nearly 800 million adults and ranking as one of the fastest-growing causes of mortality worldwide [1]. This epidemiological burden is particularly staggering in China, which currently harbors approximately 152 million affected individuals [2,3]. Although kidney transplantation remains the optimal therapeutic intervention for end-stage kidney disease (ESKD), persistent chasm between organ supply and demand poses a formidable clinical challenge [4]. Despite a national expansion in organ procurement, yielding over 24,000 solid organ transplants in 2024, an estimated 300,000 Chinese patients functionally require or await transplantation annually [5,6]. To counteract waitlist mortality, transplant centers have fundamentally shifted their acceptance criteria, increasingly relying on organs from extended-criteria donors (ECDs) and donation after circulatory death (DCD) [7,8].

However, aggressive utilization of these marginalized donor organs introduces a severe clinical tradeoff: a markedly elevated susceptibility to delayed graft function (DGF) [9,10]. Defined as the requirement for dialysis within the first postoperative week, DGF is a profound early manifestation of ischemia-reperfusion injury that currently affects 20–50% of deceased-donor kidney transplant recipients [11]. Beyond immediately extending postoperative morbidity, DGF acts as a critical biological inducer of irreversible structural damage [12]. Recent longitudinal analyses of the China Kidney Transplant Registry underscored that early DGF independently drives a 48% increased risk of chronic allograft dysfunction (CAD) over a decade of follow-up [13]. Furthermore, DGF imposes a compounding economic liability, inflating the cost of the index transplant hospitalization by approximately $18,000 and drastically extending intensive care utilization [14].

The pathophysiology of DGF is inherently multifactorial, emerging from a highly complex interplay between donor-intrinsic quality, procurement-induced ischemic damage, and recipient-specific immunological and metabolic milieus [15,16]. During preservation and reperfusion, inflammatory cascades and the release of damage-associated molecular patterns (DAMPs) precipitate severe tubular epithelial apoptosis and microvascular obstruction [17]. Despite this mechanistic understanding, traditional observational cohorts frequently conflate these overlapping variables, obscuring the distinct causal pathways that drive the initial graft failure [18]. Consequently, delineating whether DGF in a specific instance is primarily a consequence of insurmountable donor pathology or a reflection of the recipient’s heightened vulnerability remains clinically challenging [19].

Paired kidney transplantation, wherein both kidneys from a single deceased donor are allocated to two distinct recipients, provides an unparalleled quasi-experimental analytical framework to disentangle these confounding factors [20]. In instances where DGF manifests discordantly within a recipient pair, the variance in clinical outcomes can be unequivocally attributed to recipient-driven characteristics given that the donor source and initial cold ischemia parameters are virtually identical [12]. Conversely, concordant DGF, in which both recipients suffer early graft dysfunction, implicates overriding donor-related pathological determinants that eclipse individual recipient resilience [21]. Despite the immense power of this methodological design in isolating pathological drivers, studies leveraging paired kidney models remain scarce, particularly within the rapidly evolving landscape of Chinese transplant demographics.

To address this critical knowledge gap, we conducted a comprehensive retrospective analysis of 356 deceased-donor kidney transplants performed at a major Chinese transplant center between 2018 and 2022. By systematically applying a two-level analytical strategy to dissect both discordant and concordant paired outcomes, this study aimed to precisely map the bifurcated risk architecture of DGF. Delineating these distinct, quantifiable risk pathways and separating recipient-driven vulnerabilities from donor-driven deterministic factors provides a crucial evidence-based foundation for precision organ allocation. By moving beyond generalized risk algorithms, we seek to inform targeted, pathway-specific perioperative management strategies that can mitigate the dual clinical and economic burden of early allograft dysfunction, thereby optimizing the long-term utility of the increasingly vital extended-criteria donor pool.

## 2. Materials and methods

### 2.1. Study design and ethical approval

This retrospective cohort study screened all consecutive patients who underwent deceased-donor kidney transplantation at the Second Affiliated Hospital of Hainan Medical University between July 1, 2018, and October 31, 2022. The primary inclusion criterion was the receipt of a deceased-donor kidney allograft, specifically focusing on recipient pairs that received kidneys from the same deceased donor to facilitate matched within-donor analyses.

To minimize clinical confounding and ensure cohort homogeneity, patients were systematically excluded based on the following pre-specified criteria [1]: receipt of simultaneous multi-organ transplantation (e.g. combined kidney-pancreas or kidney-liver transplantation) [2]; a history of prior kidney transplantation (re-transplantation) [3]; recipients with a solitary native kidney [4]; recipients with bilateral native nephrectomy prior to transplantation [5]; recipients with a history of native kidney malignancy [6]; both kidneys from the same deceased donor transplanted into a single recipient; and [7] incomplete or missing essential donor or recipient clinical records. Following the application of these criteria, 78 patients were excluded from the initial screening pool of 434 transplants, yielding a final analytical cohort of 356 eligible recipients (82%).

### 2.2. Clinical definitions and grouping strategy

DGF was defined as the requirement for at least one hemodialysis session within the first seven days following transplantation. Donor types were classified according to the 2016 classification system proposed by Thuong et al. (Transplant International 2016;29:749-759) [22]. In our cohort, all DCD donors were controlled (type III), meaning that circulatory arrest occurred after planned withdrawal of life-sustaining therapy in the intensive care unit. No uncontrolled (type II) DCD donors were included in this study. DBCD (donation after brain death followed by circulatory death) donors correspond to ‘type IV DCD’ in the 2016 classification, defined as unexpected circulatory arrest occurring in a brain-dead donor prior to organ procurement. In this study, warm ischemia time (WIT) was defined as the ‘second warm ischemia time’, i.e. the time interval from organ retrieval from the donor to the start of cold perfusion (cold storage). This definition was applied uniformly to all donor types (DBD, DCD, and DBCD). We acknowledge that an alternative definition, ‘functional warm ischemia time’ (from withdrawal of life-sustaining therapy to cold perfusion), is typically longer for DCD and DBCD donors; however, functional WIT was not systematically recorded in our clinical database and is therefore not reported in this study. This limitation has been noted in the Discussion section. In our center, DCD and DBCD organ procurement followed the ‘rapid recovery technique’. After declaration of circulatory death and a mandatory 5-min standoff period, a rapid midline laparotomy was performed. The abdominal aorta was cannulated promptly, and in-situ cold perfusion was initiated using histidine-tryptophan-ketoglutarate (HTK) solution. Normothermic regional perfusion (NRP) was not used in any of the DCD or DBCD donors in this cohort.

To dissect the distinct contributions of recipient- and donor-driven risk factors, we applied a two-level grouping strategy. At the recipient level, individuals receiving kidneys from the same donor were classified into discordant pairs, characterized by one recipient developing DGF while the other did not. At the donor level, donors were categorized into either a concordant DGF group (both recipients developed DGF) or concordant non-DGF group (neither recipient developed DGF).

Induction therapy was administered within 30 min before transplantation based on recipient immunological risk stratification. For patients at high immunological risk (e.g. panel-reactive antibody >20% or retransplantation), antithymocyte globulin (ATG) was used at a dose of 1.5 mg/kg/day for 3–5 consecutive days. For standard-risk recipients, basiliximab was given at a dose of 20 mg intravenously within 2 h before surgery and again on postoperative day 4. Maintenance immunosuppression consisted of a triple regimen including a calcineurin inhibitor (CNI), an antiproliferative agent, and a glucocorticoid. For CNI, tacrolimus was the first-line choice, started at 0.1–0.15 mg/kg/day in two divided doses, with trough levels monitored postoperatively and maintained at 8–12 ng/mL. Cyclosporine A (4–6 mg/kg/day, trough level 150–300 ng/mL) was used as an alternative in selected cases. The antiproliferative agent was enteric-coated mycophenolate mofetil (0.72 g/day in two divided doses). For glucocorticoid therapy, methylprednisolone 500 mg was administered intraoperatively, followed by 250 mg on postoperative days 1 and 2. At discharge, oral methylprednisolone was given at 8 or 12 mg daily. In our center, all recipients received the same induction therapy regardless of donor type (DBD, DCD, or DBCD). The follow-up cutoff date was anchored to the enrollment date of the final included recipient, and the total follow-up duration was calculated from the date of individual enrollment to this cutoff to ensure standardization across the cohort.

### 2.3. Data collection

The comprehensive clinical variables were systematically extracted. Recipient-specific variables included demographic data (age, sex, and body mass index [BMI]), clinical history (duration of hypertension, diabetes mellitus, and hepatitis B surface antigen [HBsAg] status), and pre-transplant dialysis parameters (modality, duration, and immediate preoperative status). We also recorded the number of human leukocyte antigen (HLA) mismatches, baseline hematological profiles (red blood cell count, hemoglobin level, and platelet count), preoperative hemodynamics (systolic and mean arterial blood pressure), cold ischemia time (CIT), and surgical metrics (kidney laterality, operative duration, and intraoperative blood loss). Donor-specific variables included age, sex, cause of death, cardiopulmonary resuscitation history, terminal serum creatinine level, BMI, hypertension, diabetes mellitus, CIT, and warm ischemia time (WIT).

### 2.4. Statistical analysis

Continuous variables were first assessed for normality. Normally distributed continuous data were expressed as mean ± standard deviation (SD), whereas non-normally distributed data were reported as medians with interquartile ranges (IQR). Categorical variables are summarized as counts and percentages. Between-group clinical differences were evaluated using independent-samples *t*-tests or Mann-Whitney *U* tests for continuous parameters and Pearson’s *χ^2^* or Fisher’s exact tests for categorical data.

**Figure 1.**
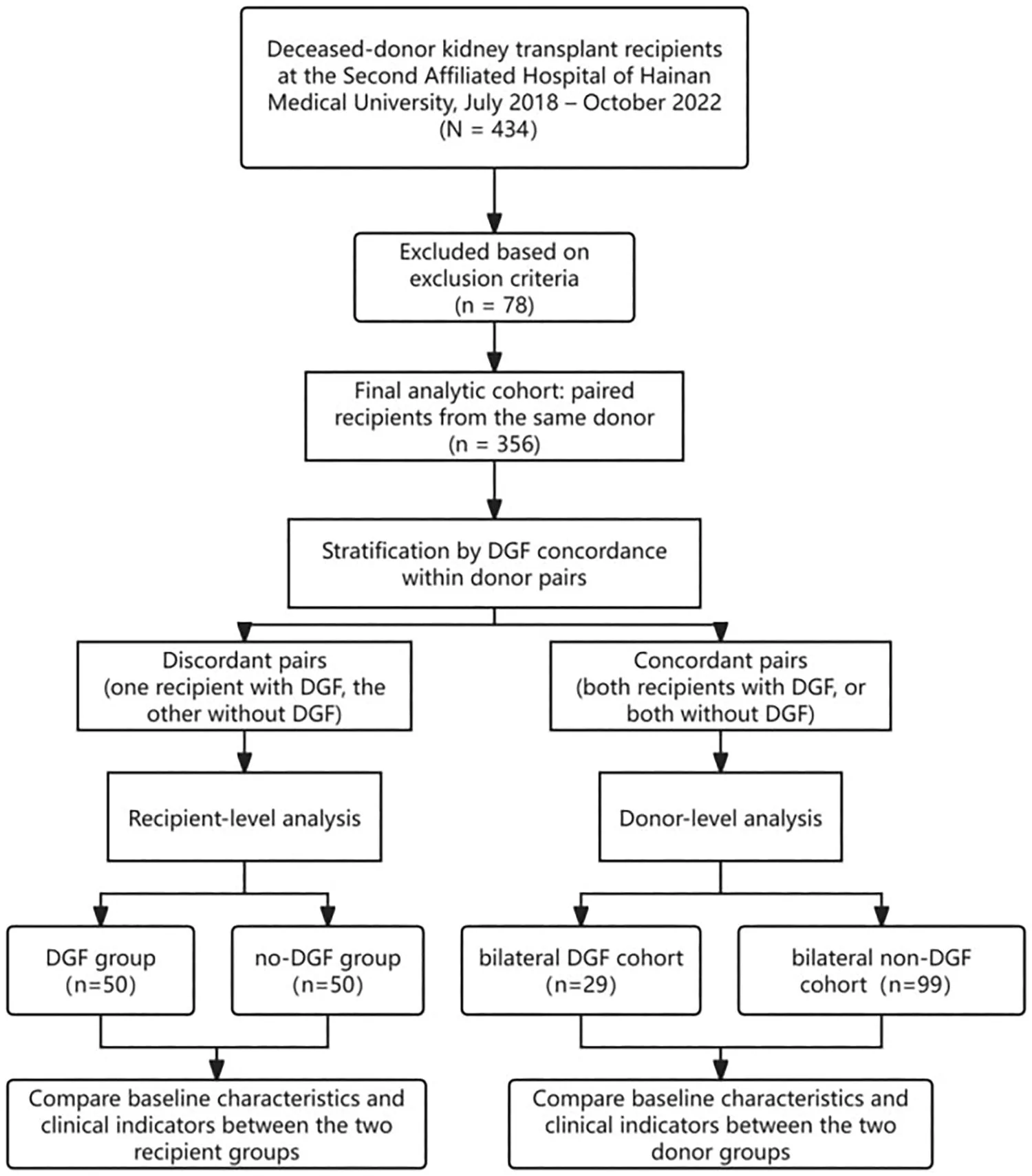
Study design flowchart.

To isolate independent predictors of DGF, multivariable logistic regression models were constructed, with results presented as regression coefficients (β), standard errors (SE), Wald *χ^2^* statistics, odds ratios (OR), and 95% confidence intervals (CI). For overall survival (OS) analyses, a univariate Cox proportional hazards regression model was initially employed. Given the low incidence of mortality events in this cohort (*n* = 5), a relaxed significance threshold of *p* < 0.10 was prespecified for univariable screening to optimize signal detection. Continuous variables achieving this threshold were dichotomized into high and low strata by identifying the optimal cutoff values that maximized the log-rank statistic. Survival distributions were estimated using the Kaplan–Meier method, visually represented with risk tables, and compared statistically using the log-rank test. The median survival time was calculated for each subgroup. Variables significant in the univariable analysis were subsequently incorporated into a multivariable Cox regression model, with associations reported as hazard ratios (HR) and 95% CIs. For all other primary analyses, a two-sided *p* value of < 0.05 was considered statistically significant. A detailed study design flowchart is presented in Figure 1.

## 3. Results

### 3.1. Study cohort and recipient grouping strategy

During the designated study period, an initial cohort of 434 consecutive patients who underwent deceased-donor kidney transplantation was evaluated. To ensure cohort homogeneity and minimize clinical confounding, we systematically excluded 78 cases involving multi-organ transplantation, re-transplantation, major underlying clinical comorbidities, or incomplete data records. Consequently, the final analytical cohort comprised of 356 eligible recipients, representing 82% of the initially screened population. To rigorously dissect recipient-specific versus donor-specific drivers of DGF, we stratified the cohort according to DGF occurrence within paired recipients receiving kidneys from the same donor. At the recipient level, we isolated a discordant subcohort consisting of a DGF group (*n* = 50) and a paired no-DGF group (*n* = 50), allowing us to assess recipient vulnerabilities while inherently controlling for donor organ quality. At the donor level, the donors were stratified into a bilateral DGF cohort (where both paired recipients developed DGF; *n* = 29) and a bilateral non-DGF cohort (where neither paired recipient developed DGF; *n* = 99).

### 3.2. Recipient-Driven predictors of discordant DGF

First, we evaluated the baseline demographic and clinical indicators distinguishing the recipient DGF group from the paired no-DGF group to identify specific recipient-driven vulnerabilities (Table **1**). Univariable comparisons of continuous variables revealed significant metabolic and immunological disparities between cohorts. Notably, the mean BMI was significantly elevated in the DGF group compared to the no-DGF group (22.39 ± 2.78 vs. 21.07 ± 3.00 kg/m^2^, *p* = 0.024). Furthermore, the pre-transplant duration of recipient hypertension was significantly longer in the DGF cohort (median 4.00 vs. 3.00 years, *p* = 0.015), as was the duration of pre-transplant dialysis (median 3.00 vs. 2.00 years, *p* = 0.008). Immunologically, recipients who developed DGF exhibited a significantly higher number of HLA mismatches than their DGF-free counterparts (median 4.00 vs. 3.00, *p* = 0.002). Surgical parameters also diverged, with intraoperative blood loss being substantially greater in the DGF group (median 175.00 vs. 100.00 mL, *p* = 0.018).

**Table 1.** Comparison of recipient baseline characteristics and clinical indicators between the No DGF and DGF groups.

Item	No DGF group (*N* = 50)	DGF group (*N* = 50)	*p-*Value
Age (years)	42.08 ± 12.07	42.20 ± 13.56	0.963
Female	17 (34%)	11 (22%)	0.265
BMI (kg/m^2^)	21.07 ± 3.00	22.39 ± 2.78	0.024
HBsAg-positive state	9 (18%)	8 (16%)	1.000
Hypertension (years)	3.00 (1.00, 5.00)	4.00 (2.00, 8.00)	0.015
Diabetes Mellitus	6 (12%)	10 (20%)	0.413
Dialysis modality			0.246
No dialysis	0 (0%)	1 (2%)	
Hemodialysis	34 (68%)	34 (68%)	
Peritoneal dialysis	13 (26%)	15 (30%)	
Mixed dialysis	3 (6%)	0 (0%)	
Dialysis time (years)	2.00 (1.00, 3.00)	3.00 (2.00, 5.00)	0.008
HLA Mismatches	3.00 (2.00, 4.00)	4.00 (3.00, 5.00)	0.002
RBC (10⁶/μL)	3.80 ± 0.75	3.77 ± 0.86	0.852
Hb (g/L)	112.50 (91.00, 123.00)	113.50 (99.00, 129.00)	0.567
Platelets (10³/μL)	206.00 (173.00, 252.00)	192.50 (166.00, 227.00)	0.184
Preoperative dialysis			0.531
No dialysis	36 (72%)	31 (62%)	
Hemodialysis	9 (18%)	11 (22%)	
Peritoneal dialysis	5 (10%)	8 (16%)	
SBP (mmHg)	144.96 ± 17.94	138.80 ± 17.62	0.086
DBP (mmHg)	84.00 (77.00, 100.00)	85.00 (77.00, 92.00)	0.898
MAP (mmHg)	105.20 ± 15.12	103.76 ± 13.88	0.621
CIT (hours)	3.50 (3.00, 5.00)	4.00 (3.00, 6.00)	0.713
Transplanted kidney			0.841
Left Kidney	24 (48%)	26 (52%)	
Right Kidney	26 (52%)	24 (48%)	
Duration of surgery (min)	162.38 ± 43.87	178.32 ± 42.92	0.069
Intraoperative Blood loss (mL)	100.00 (50.00, 200.00)	175.00 (100.00, 200.00)	0.018

Abbreviations: DGF, delayed graft function; BMI, body mass index; HBsAg, hepatitis B surface antigen; HLA, human leukocyte antigen; RBC, red blood cell; Hb, hemoglobin; Plt, platelet; SBP, systolic blood pressure; DBP, diastolic blood pressure; MAP, mean arterial pressure; CIT, cold ischemia time.

Conversely, no statistically significant between-group differences were observed across other continuous hemodynamic and operative variables, including recipient age, preoperative red blood cell count, hemoglobin level, platelet count, systolic and diastolic blood pressures, mean arterial pressure, CIT, and total operative duration (all *p* > 0.05). Similarly, categorical variables, including recipient sex, HBsAg positivity, diabetes mellitus prevalence, specific dialysis modality, immediate preoperative dialysis status, and transplanted kidney laterality, demonstrated no significant distributional disparities between the two groups (all *p* > 0.05). Guided by these univariable screening results, higher BMI, prolonged hypertension and dialysis duration, an increased number of HLA mismatches, and greater intraoperative blood loss were identified as candidate risk factors for advanced multivariable modeling.

To determine the independent predictive value of these recipient-specific factors for early graft outcomes, we constructed a multivariable logistic regression model encompassing BMI, duration of hypertension, dialysis duration, number of HLA mismatches, and intraoperative blood loss. After rigorous adjustment for these covariates, three variables emerged as robust independent predictors of DGF (Table 2). Recipient BMI remained significantly associated with DGF risk (odds ratio [OR] = 1.22, 95% confidence interval [CI] 1.03–1.44; *p* = 0.019). Additionally, prolonged pre-transplant dialysis duration (OR = 1.31, 95% CI 1.01–1.69; *p* = 0.041) and an increased number of HLA mismatches (OR = 1.47, 95% CI 1.06–2.02; *p* = 0.019) were independently correlated with an increased likelihood of early graft dysfunction. In contrast, the pre-transplant duration of hypertension (OR = 1.08, 95% CI 0.96–1.20; *p* = 0.186) and the volume of intraoperative blood loss (OR = 1.36, 95% CI 0.92–2.00; *p* = 0.117) were not significantly associated with DGF in the multivariable analysis.

**Table 2.** Risk factor analysis for recipient DGF outcome (multivariate).

Item	*β*	*SE*	Wald *χ*²	OR (95% CI)	*p-*Value
BMI (kg/m^2^)	0.198	0.084	5.49	1.22 (1.03, 1.44)	0.019
Hypertension (years)	0.074	0.056	1.748	1.08 (0.96, 1.20)	0.186
Dialysis time (years)	0.267	0.131	4.17	1.31 (1.01, 1.69)	0.041
HLA Mismatches	0.383	0.163	5.508	1.47 (1.06, 2.02)	0.019
Blood loss (mL)	0.309	0.197	2.452	1.36 (0.92, 2.00)	0.117

Abbreviations: BMI, body mass index; HLA, human leukocyte antigen; OR, odds ratio; CI, confidence interval.

To address the potential confounding effect of residual native renal function, we performed sensitivity analyses excluding recipients with ESRD duration < 2 years (*n* = 69) and < 1 year (*n* = 92). In both sensitivity analyses, BMI (OR = 1.40, 95% CI 1.09–1.78, *p* = 0.008; OR = 1.28, 95% CI 1.06–1.54, *p* = 0.010) and HLA mismatches (OR = 1.58, 95% CI 1.01–2.46, *p* = 0.046; OR = 1.55, 95% CI 1.09–2.21, *p* = 0.015) remained significant predictors of DGF. Dialysis duration remained significant in the ESRD ≥ 1 year subset (OR = 1.39, 95% CI 1.04–1.86, *p* = 0.026), with a stable effect size in the ESRD ≥ 2 years subset (OR = 1.32, 95% CI 0.90–1.94) but reduced statistical power due to the smaller sample size. These findings indicate that residual native renal function is unlikely to have substantially confounded our primary results.

### 3.3. Post-Transplant recipient survival outcomes

We subsequently assessed the impact of these clinical parameters on post-transplant overall survival (OS) through univariate and multivariate Cox proportional hazards regression analyses (Table 3). Due to the inherently low incidence of post-transplant mortality events in our cohort (*n* = 5), we prespecified a relaxed significance threshold of *p* < 0.10 for the univariable screening phase to maximize the detection of potential survival associations. In the univariate models, both recipient BMI and age met this threshold. BMI demonstrated the most prominent univariable association with mortality risk (hazard ratio [HR] = 1.43, 95% CI 1.02–2.01; *p* = 0.037), while age exhibited a borderline association (HR = 1.08, 95% CI 0.99–1.17; *p* = 0.070). Other clinical and operative variables failed to meet the *p* < 0.10 inclusion criterion. Furthermore, hazard ratios for several categorical variables, such as recipient sex, HBsAg status, and preoperative dialysis status, could not be reliably computed because of complete data separation, as all recorded mortality events were clustered within specific demographic subgroups. When BMI and age were co-entered into a multivariable Cox regression framework, only BMI maintained its independent predictive value for post-transplant OS at the prespecified threshold (HR = 1.38, 95% CI 0.95–2.00; *p* = 0.090). The association between recipient age and OS was attenuated and lost statistical significance upon multivariate adjustment (HR = 1.06, 95% CI 0.98–1.15; *p* = 0.164).

**Table 3.** Overall survival (OS) in recipients following transplantation.

	*Univariate*		*Multivariate*	
Item	HR (95%CI)	*p-*Value	HR (95%CI)	*p-*Value
BMI (kg/m^2^)	1.43 (1.02, 2.01)	0.037	1.38 (0.95, 2.00)	0.090
Age (years)	1.08 (0.99, 1.17)	0.070	1.06 (0.98, 1.15)	0.164
Blood loss (mL)	1.00 (1.00, 1.01)	0.105		
SBP (mmHg)	0.96 (0.90, 1.02)	0.182		
HLA Mismatches	0.64 (0.34, 1.23)	0.185		
Dialysis time (years)	0.71 (0.41, 1.24)	0.232		
Transplanted kidney (Left/Right)	0.27 (0.03, 2.40)	0.239		
CIT (hours)	0.79 (0.50, 1.25)	0.316		
RBC (10⁶/μ L)	0.68 (0.25, 1.82)	0.442		
Hb (g/L)	0.99 (0.95, 1.02)	0.442		
Duration of surgery (min)	1.01 (0.99, 1.03)	0.458		
DGF	1.58 (0.26, 9.44)	0.618		
MAP (mmHg)	0.99 (0.94, 1.05)	0.714		
Hypertension (years)	1.04 (0.83, 1.32)	0.724		
Diabetes Mellitus	1.38 (0.15, 12.37)	0.775		
DBP (mmHg)	1.00 (0.96, 1.05)	0.849		
Platelts (10³/μL)	1.00 (0.99, 1.01)	0.918		
Preoperative dialysis	0.92 (0.10, 8.27)	0.941		

Abbreviations: BMI, body mass index; HLA, human leukocyte antigen; HR, hazard ratio; CI, confidence interval; RBC, red blood cell; Hb, hemoglobin; Plt, platelet; SBP, systolic blood pressure; MAP, mean arterial pressure; DBP, diastolic blood pressure; CIT, cold ischemia time; DGF, delayed graft function.

**Figure 2.**
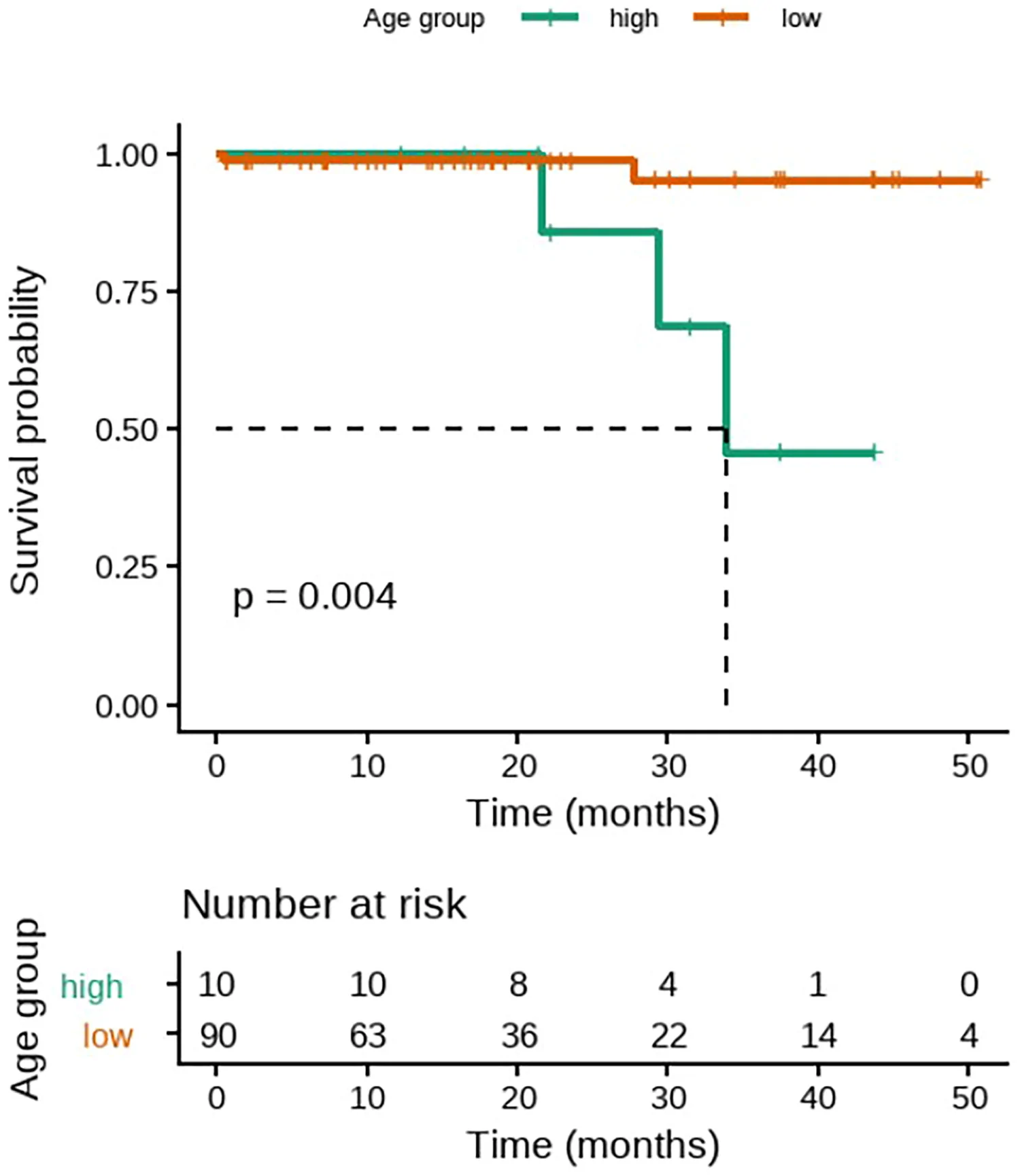
Kaplan-Meier Survival Curve Stratified by Recipient Age (>59 Years vs. ≤59 Years).

**Figure 3.**
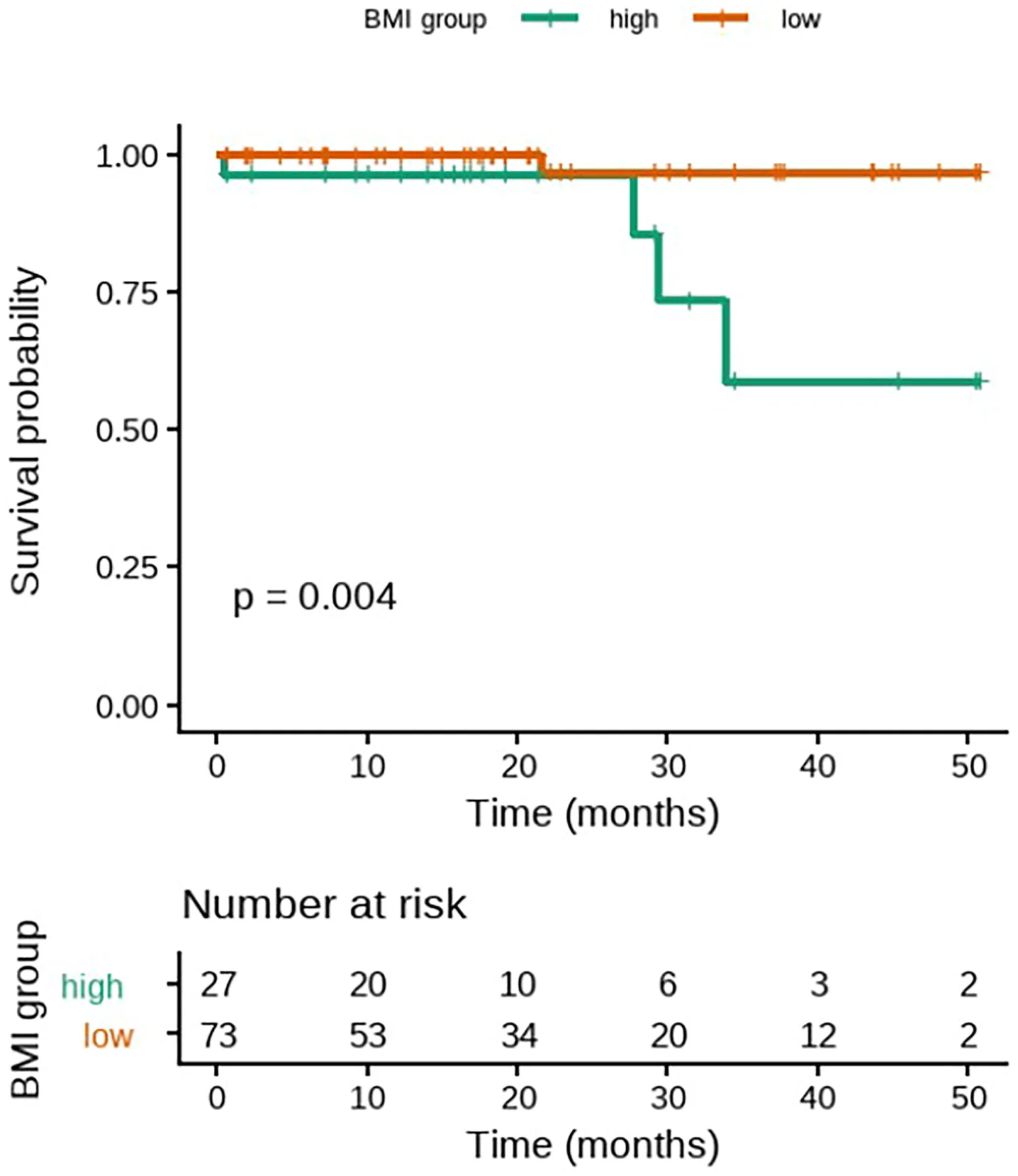
Kaplan-Meier Survival Curve Stratified by Body Mass Index (BMI) (>23.43 vs. ≤23.43).

Kaplan-Meier survival estimations were performed to dynamically characterize survival trajectories relative to recipient age and BMI. Continuous variables were dichotomized into high- and low-risk strata, utilizing optimal cutoff values determined by maximizing the log-rank statistic. Across the entire cohort, the median follow-up duration was 18.3 months (95% CI 16.4-22.0), with the vast majority of recipients monitored for over 18 months post-transplantation. Applying a statistically optimized age cutoff of 59 years, the older cohort (>59 years; *n* = 10) experienced three mortality events, yielding a median survival of 33.9 months (95% CI 29.4-not reached). Conversely, the younger cohort (≤59 years; *n* = 90) had only two deaths, and their median survival remained unreached. Log-rank testing confirmed a significant decrease in survival in the older recipient demographics (*χ*^2^ = 8.27, *df* = 1; *p* = 0.004) (Figure 2). A parallel analysis for BMI utilized an optimal cutoff of 23.43 kg/m^2^. The high-BMI cohort (>23.43 kg/m^2^; *n* = 27) registered 4 mortality events with a median survival of 29.4 months (95% CI 29.4–not reached), whereas the low-BMI group (≤23.43 kg/m^2^; *n* = 73) experienced a single mortality event, with median survival not reached. This divergence translated into a statistically significant reduction in OS for recipients with an elevated BMI (*χ*^2^ = 8.29, df = 1; *p* = 0.004) (Figure 3). Among the five mortality events, four were attributed to pulmonary infection and one to other causes. All five deaths occurred in patients with a functioning graft, and no patient returned to dialysis prior to death. Therefore, the survival differences observed in the Kaplan-Meier analyses (Figures 1 and 2) reflect deaths with a functioning graft rather than graft failure leading to death. The predominance of infection-related deaths in this cohort underscores the potential role of post-transplant infectious complications, which may be influenced by recipient BMI and age.

### 3.4. Donor-level drivers of concordant bilateral DGF

By shifting the analytical focus from recipient susceptibility to intrinsic allograft quality, we investigated donor factors associated with concordant bilateral DGF (Table 4). The donor cohort consisted of 128 deceased donors, of whom 99 (77.3%) were classified into the bilateral non-DGF group and 29 (22.7%) into the bilateral DGF group. Univariable profiling revealed significant discrepancies in the baseline donor quality metrics between the two cohorts. Donors whose kidneys precipitated bilateral DGF were significantly older (*p* = 0.017) and had a higher incidence of preceding cardiopulmonary resuscitation (*p* = 0.019) than donors in the bilateral no-DGF group. Crucially, the bilateral DGF donor cohort was characterized by severe functional and chronic end-organ damage, manifesting as markedly elevated terminal serum creatinine levels (*p* < 0.001), longer preexisting durations of donor hypertension (*p* < 0.001) and diabetes mellitus (*p* = 0.022), and significantly extended WIT (*p* < 0.001). Additionally, aggregated recipient-level parameters for these specific donor pairs demonstrated a higher burden of HLA mismatches (*p* < 0.001) and longer operative durations (*p* = 0.003). Other general donor factors, such as sex, specific cause of death, BMI, and CIT, as well as other paired recipient parameters, showed no statistically significant univariable differences (all *p* > 0.05).

**Table 4.** Comparison of donor baseline characteristics and clinical indicators between the concordant No DGF and concordant DGF groups(univariate).

Item	No DGF (*N* = 99)	DGF (*N* = 29)	*p-*Value
Female	22 (22.2%)	6 (20.7%)	1.000
Age (years)	47.00 (39.00,53.50)	54.00 (45.00,58.00)	0.017
Form of death			0.410
DCD	27 (27.3%)	10 (34.5%)	
DBD	58 (58.6%)	13 (44.8%)	
DBCD	14 (14.1%)	6 (20.7%)	
Cardiopulmonary resuscitation status	35 (35.4%)	18 (62.1%)	0.019
Terminal creatinine (mg/dL)	0.60 (0.46, 0.71)	0.96 (0.61, 1.65)	<.001
BMI (kg/m^2^)	22.03 ± 2.68	23.22 ± 3.66	0.112
Hypertension duration (years)	0.00 (0.00, 0.00)	2.00 (0.00, 3.00)	<.001
Diabetes duration (years)	0.00 (0.00, 0.00)	0.00 (0.00, 1.00)	0.022
CIT (hours)	3.00 (2.50, 4.00)	4.00 (3.00, 4.50)	0.200
WIT (min)	4.00 (3.00, 5.00)	5.00 (4.00, 6.00)	<.001

*Note*. WIT in this table refers to second warm ischemia time (from organ retrieval to cold perfusion).

Abbreviations: DGF, delayed graft function; DCD, donation after circulatory death; DBD, donation after brain death; DBCD, donation after brain death followed by circulatory death; BMI, body mass index; CIT, cold ischemia time; WIT, warm ischemia time. (WIT in this table refers to second warm ischemia time, from organ retrieval to cold perfusion.)

To isolate the definitive donor-derived drivers capable of dictating bilateral DGF irrespective of recipient variations, variables achieving univariable significance were subjected to a multivariable logistic regression model optimized *via* the Bayesian Information Criterion (BIC) (Table 5). The final model demonstrated that intrinsic graft injury parameters affected the general donor demographic traits. Specifically, elevated donor terminal creatinine (OR = 1.03, 95% CI 1.01–1.05; *p* = 0.001), prolonged duration of donor hypertension (OR = 2.40, 95% CI 1.52–3.81; *p* < 0.001), and extended warm ischemia time (WIT; OR = 1.67, 95% CI 1.15–2.44; *p* = 0.007) were identified as powerful independent predictors of concordant bilateral DGF. Conversely, broader donor risk markers that were significant in univariable screening, namely, age, cardiopulmonary resuscitation history, body weight, and diabetes mellitus, were completely attenuated and not retained in the final multivariable model. This analytical pattern indicates that the apparent risk conferred by these general systemic factors is likely mediated through, or fundamentally correlated with, direct parenchymal and microvascular damage captured by elevated terminal creatinine, chronic hypertensive nephropathy, and prolonged ischemic injury.

**Table 5.** Risk factor analysis for donor DGF outcome (multivariate).

Item	*β*	*SE*	Wald *χ*²	OR (95% CI)	*p-*Value
Terminal creatinine (mg/dL)	0.028	0.009	3.286	1.03 (1.01, 1.05)	0.001
Hypertension (years)	0.877	0.235	3.73	2.40 (1.52, 3.81)	<0.001
WIT (min)	0.515	0.192	2.685	1.67 (1.15, 2.44)	0.007

Abbreviations: WIT, warm ischemia time; OR, odds ratio; CI, confidence interval.

## 4. Discussion

Although the risk factors for DGF following deceased-donor kidney transplantation have been extensively studied, studies dissecting the relative contributions of donor versus recipient variables remain notably sparse, particularly within Chinese cohorts. By leveraging a paired kidney allocation model, in which two kidneys from a single deceased donor were transplanted into distinct recipients, we established a rigorous analytical framework to isolate these effects. Our two-tiered approach revealed that discordant DGF outcomes are independently driven by recipient-specific vulnerabilities, namely higher BMI, prolonged pre-transplant dialysis, and greater HLA mismatching. Conversely, concordant (bilateral) DGF is predominantly dictated by donor-derived insults, specifically elevated terminal creatinine levels, chronic donor hypertension, and prolonged WIT. These findings underscore the complex interplay between baseline allograft quality and recipient-specific susceptibility to ischemia-reperfusion injury [23].

In our analysis of discordant pairs, elevated recipient BMI emerged as a robust independent predictor of early graft dysfunction [24]. This clinical observation aligns closely with the ‘nephron underdosing’ hypothesis, which posits that the heightened metabolic and filtration demands of obese recipients precipitate an allostatic load that is poorly tolerated by a newly implanted, ischemic allograft [25]. Furthermore, obesity introduces technical surgical complexities that prolong the operative duration. Beyond hemodynamics and surgical logistics, the chronic low-grade inflammatory state endemic to adiposity, characterized by the constitutive systemic release of proinflammatory cytokines such as IL-6 and TNF-α, may synergistically amplify innate immune activation triggered by ischemia-reperfusion injury [26]. Although our survival analyses suggest an association between higher BMI and post-transplant mortality must be interpreted cautiously due to limited event rates, these mechanistic links provide a compelling rationale for aggressive pre-transplant weight management and nutritional optimization.

The recipient’s pre-transplant metabolic and immunological milieu further dictates the trajectory of functional graft recovery. We identified prolonged dialysis duration as a critical vulnerability, consistent with its established role as a major driver of cardiovascular morbidity and overall mortality. Extended dialysis regimens serve as a clinical surrogate for profound uremic burden, precipitating systemic endothelial dysfunction, vascular calcification, and intractable chronic inflammation [27]. Mechanistically, this compromised endothelial landscape impairs vascular autoregulation and repair capacity upon reperfusion, thereby magnifying the ischemic injury within the graft. Furthermore, the accumulation of uremic toxins—including indoxyl sulfate, p-cresyl sulfate, and advanced glycation end-products—induces oxidative stress and promotes a pro-inflammatory state that may sensitize the allograft to ischemia-reperfusion injury [28]. These toxins activate the aryl hydrocarbon receptor (AhR) pathway and upregulate pro-inflammatory cytokines such as TNF-α and IL-6, which can directly impair tubular epithelial cell viability and exacerbate the inflammatory response triggered by reperfusion [29]. The duration of dialysis exposure serves as a cumulative measure of this uremic burden, with each additional year of dialysis contributing to progressive vascular stiffening, medial calcification, and reduced microvascular density—all of which compromise the graft’s ability to withstand the hemodynamic and oxidative stresses of reperfusion [30]. Our findings suggest that even after successful transplantation, this preexisting vascular pathology may predispose recipients to delayed functional recovery, highlighting the importance of minimizing dialysis vintage through timely transplant referral and preemptive transplantation strategies. Parallel to this metabolic deterioration is the profound impact of alloreactivity; we found that a higher degree of HLA mismatching independently precipitated DGF [31,32]. Distinct from classic early acute cellular rejection, this phenomenon suggests that alloantigen-driven adaptive immune responses act rapidly to exacerbate innate ischemic tissue injury, severely retarding functional recovery [33,34]. Consequently, incorporating rigorous HLA matching algorithms and prioritizing patients with shorter dialysis vintages may be paramount for minimizing early graft dysfunction [35].

While recipient variables modulate the individual response to a given graft, our analysis of concordant DGF unequivocally demonstrates that severe donor-derived insults can override recipient-level resilience [36–38]. Donor terminal creatinine level, longstanding donor hypertension, and prolonged WIT were independently associated with bilateral DGF. These parameters, particularly hypertension and terminal creatinine, are fundamental elements of the Kidney Donor Profile Index (KDPI), a validated metric for intrinsic allograft quality [39]. Prolonged WIT directly induces severe cellular hypoxia, ATP depletion, and subsequent oxidative stress upon reperfusion, serving as the primary biological driver of ischemic tissue necrosis [40,41]. Our data suggest a pathogenic threshold: once the cumulative burden of donor organ injury and ischemia exceeds a critical limit, the resultant structural damage exerts a dominant deterministic effect on early graft function. Under these conditions, the probability of DGF becomes uniformly high across both recipients, effectively eclipsing specific recipient characteristics [42,43]. This necessitates a precision-medicine approach to organ allocation; high-risk kidneys with these characteristics should ideally be allocated to highly resilient recipients lacking additional metabolic or immunological comorbidities to blunt the compounded risk.

The interpretation of our findings must be contextualized within several methodological limitations. Primarily, this was a single-center, retrospective analysis with a relatively restricted sample size, lacking validation across larger, multi-institutional registries. The relatively short follow-up duration and low incidence of hard endpoints (death events) fundamentally restrict our statistical power, precluding the robust estimation of hazard ratios across several categorical subgroups. Our survival analysis was limited by the small number of mortality events (*n* = 5). All observed deaths were deaths with a functioning graft, with pulmonary infection being the leading cause (4/5). Death-censored graft survival could not be reliably analyzed due to the absence of graft failure requiring dialysis return in this cohort. Therefore, the survival associations with BMI and age should be interpreted as overall mortality risk, which in our cohort was primarily driven by infectious complications rather than graft dysfunction. Furthermore, our study timeline intersected with the COVID-19 pandemic, which may have influenced perioperative management, post-transplant infection risk, and long-term survival outcomes. Although we could not directly quantify its impact, the pandemic represents an unmeasured systemic confounder that should be considered when interpreting the high proportion of infection-related deaths in this cohort. Moreover, our multivariable models were limited by the available retrospective data; unmeasured variables of high clinical relevance, such as the specific etiologies of primary renal disease, were not incorporated and may harbor residual confounding effects. Additionally, functional WIT for DCD/DBCD donors was not systematically recorded in our database, and we therefore reported only second WIT in this study. Finally, our definition of DGF (requirement for dialysis within 7 days post-transplant) did not exclude dialysis performed for non-graft dysfunction indications such as hyperkalemia, volume overload, or other metabolic derangements, which may lead to potential misclassification. While this definition aligns with standard clinical practice and facilitates comparability across studies, we acknowledge that it may not fully differentiate between true graft failure and dialysis required for other clinical indications. Additionally, panel reactive antibody (PRA) data were not systematically available for all recipients in this retrospective cohort, as PRA testing in our center is performed selectively for patients with prior sensitizing events rather than as a universal screening tool. This represents a limitation of the data source.

In conclusion, by utilizing a paired kidney model, this study successfully bifurcated distinct donor- and recipient-driven pathways culminating in DGF. We provide actionable clinical insights: donor organs exhibiting elevated terminal creatinine, chronic hypertensive damage, and prolonged warm ischemia demand a highly selective allocation. Conversely, transplant candidates burdened by a high BMI, prolonged dialysis dependence, and significant HLA mismatches require intense pre-habilitation and tailored perioperative management. Integrating these targeted and stratified approaches into routine clinical pathways holds substantial promise for mitigating the incidence of DGF and extending allograft longevity.

## Data Availability

The datasets used and/or analyzed during the current study are available from the corresponding author upon reasonable request.

## Submission declaration and verification

We confirm that this manuscript contains novel research findings that have not been previously published and are not currently under consideration elsewhere. All authors have thoroughly reviewed and agreed to submit this manuscript for publication.
